# A New Approach to Decoupled Non-Resonant Polishing

**DOI:** 10.3390/mi10070484

**Published:** 2019-07-18

**Authors:** Yucheng Li, Xiaoqin Zhou, Guilian Wang, Peiqun Ma, Rongqi Wang

**Affiliations:** 1 School of Mechanical and Aerospace Engineering, Jilin University, Changchun 130022, China; 2 Tianjin Key Laboratory for Advanced Mechatronic System Design and Intelligent Control, Tianjin University of Technology, Tianjin 300384, China; 3 Shanghai Automotive Industry Corporation (Group) Motor Technical Center, Shanghai 201804, China

**Keywords:** two-dimensional vibration-assisted polishing (2D-VAP), decoupled, removal function

## Abstract

Two-dimensional vibration-assisted polishing (2D-VAP) is a new polishing approach proposed in recent years, which is considered to be very suitable for the polishing of micro-optical parts and micro-structured surfaces. The advantages of the 2D-VAP approach are as follows: A very high relative velocity can be obtained between the workpiece and the polishing tool; the desired motion trajectory can be formed in one polishing cycle. However, there are still some problems to be solved in the 2D-VAP approach, mainly involving: The vibration frequency of the resonant excitation cannot be regulated, which makes it difficult to adapt to the processing demands of different materials; the theoretical model of removal function has been studied in few papers; and motion coupling occurs easily between the horizontal and vertical directions, which affects the trajectory synthesized at the polishing tool. In order to solve these problems, a new approach to decoupled non-resonant polishing is developed in this paper, and its effectiveness is investigated by the theoretical analysis and polishing experiments. Theoretical studies of removal function show that the vibration frequency, vibration amplitude and loading force are proportional to the removal depth. The comparison of experimental and modeling results of removal function show that they have good coherence, and the correctness of the theoretical model of removal function is verified. In addition, the stability experiments of removal function prove that the polishing approach has better stability and is beneficial to the convergence of workpiece surface.

## 1. Introduction

Smooth and ultra-smooth surfaces are widely used in various engineering fields, such as aerospace engineering, medical equipment, optical telecommunication, nanotechnology and microelectronics. Therefore, processing technology of smooth and ultra-smooth surfaces should be highly efficient and stable. It has become an important topic of research in precision machining, and it has attracted more and more attention. The time of processing is uncertain in the traditional ways of contact type polishing, and scratches on the polished surface will easily be found. It is difficult to meet the requirements of smooth and ultra-smooth surfaces in the development of modern science. Therefore, international academia and industry made a large quantity of theories and experimental researches to break through the traditional ways of polishing and put forward innovative ways of polishing in the recent past, such as magneto-rheological finishing [1,2,3], plasma assisted polishing [4,5], fluid jet polishing [6,7,8], two dimensional vibration-assisted polishing and other composite polishing methods [9,10,11,12].

In recent years, more and more scholars have focused on vibration-assisted processing technology, mainly including vibration-assisted cutting [13,14,15,16], vibration-assisted grinding [17,18,19,20] and vibration-assisted polishing [21,22,23,24,25,26,27,28]. Vibration-assisted polishing technology is mainly divided into resonant mode and non-resonant mode. In terms of non-resonant mode researches, Chee et al. put forward two non-resonant polishing methods, and both of them were verified by experiments [25,26]. Guo et al. proposed a magnetostrictive vibration polishing device, which had the advantages of miniaturization, low-voltage driving and high power. The polishing process was completed by the interaction of 5-axis Computer Numerical Control (CNC) machine tool and a real-time polishing force control system [27,28].

The advantages of non-resonant mode polishing devices are as follows: The vibration frequency of polishing tool can be adjusted autonomously; a large amount of heat can be avoided during processing due to the relatively low vibration frequency. But there are still some problems of non-resonant mode polishing devices; the motion coupling in the synthetic trajectory of the polishing tool is one of the problems in the process. It will affect the shape of the motion trajectory synthesized at the polishing tool, which will influence the polishing effect. However, it was often overlooked in 2D-VAP and was not considered in the previous vibration-assisted polishing studies. It is also worth noting that the removal function plays an important role in the vibration-assisted polishing result, which is directly related to whether the surface of the workpiece is converged after polishing. Therefore, it is necessary to study the removal function from theory and experiment.

For the above problems, the following improvements have been made on the basis of the device proposed by our research group in the paper [29]. The flexure hinge mechanism with symmetric distribution is adopted in the device proposed in this paper, with which the coupling phenomenon at the polishing tool in the vibration polishing process can be effectively avoided. The decoupling of the flexure hinge mechanism was verified by the offline performance test. Furthermore, the straight beam-type flexure hinge is improved to be half corner-filleted flexure hinge, which has the advantages of high precision, large range of motion and greater turning ability. The connection between the half corner-filleted flexure hinges and the central rigid platform is chamfered to prevent stress concentration and generating fatigue cracks. According to the polishing process and the piezoelectric driving mode, the polishing force adjustment mechanism was also designed to realize the processing of constant polishing force. Few researchers have studied the theoretical model of removal function with the circular polishing trajectory in the previous non-resonant vibration-assisted polishing studies. The details of the theoretical model of removal function and simulation analyses under different parameters were carried out in this paper. In addition, the correctness of the theoretical model of the removal function was verified by comparing the modeling and experimental results. Due to the importance of the stability of removal function, and was rarely studied in the vibration-assisted polishing in the previous studies. Therefore, the stability of removal function was studied in the experimental part, and the feasibility and stability of the polishing system proposed in this paper were verified by it.

## 2. Mechanism Design

### 2.1. Principle of Decoupled Non-Resonant Two-Dimensional Vibration-Assisted Polishing (2D-VAP)

**Figure 1:**
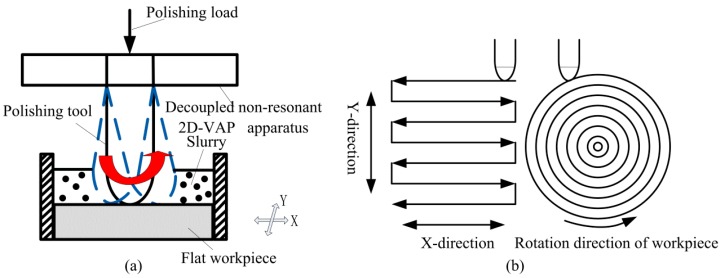
Diagram of vibration-assisted polishing. (**a**) Schematic diagram of material removal by using motion trajectory formed by the flexure mechanism; (**b**) removal of the surface material according to the raster and concentric circle path.

The decoupled non-resonant 2D-VAP device is driven by stacked piezoelectric actuators directly to the flexure hinges, which make the flexure hinges deformed. Thereby driving the polishing tool to generate the motion trajectory of a certain vibration frequency and a certain vibration amplitude so as to achieve the deterministic removal of surface material. Figure 1a is a schematic diagram of material removal by using the closed motion trajectory formed through the flexure hinges driven by stacked piezoelectric actuators. Under the precondition of Figure 1a, Figure 1b shows the removal of the surface material according to the specified polishing path by the polishing tool. The polishing paths in Figure 1b are two common polishing paths, the raster path and the concentric circle path, respectively. The workpiece is driven by the machine tool to reciprocate in the horizontal direction to realize the raster path. The concentric circular path is achieved according to that the polishing tool moves at a certain feed speed when the workpiece is rotated.

The vibration of the decoupled non-resonant 2D-VAP in the X-direction and Y-direction can be expressed as:

(1) $$\left\{ \begin{array}{l} x=A_{1}\sin\left(2\pi f_{1}t+\theta_{1}\right)\\ y=A_{2}\sin\left(2\pi f_{2}t+\theta_{2}\right) \end{array}\right.$$

where $A_{1}$, $A_{2}$, $f_{1}$, $f_{2}$, $\theta_{1}$ and $\theta_{2}$ are the vibration amplitudes, vibration frequencies and initial phase in the X-direction and Y-direction, respectively; $\Delta \theta =\theta_{2}-\theta_{1}$ is the phase shift between the X-direction and Y-direction, and *t* is the time variation. By changing the parameters in the vibration expression, different shapes of polishing trajectories can be achieved.

### 2.2. Structure Design

**Figure 2:**
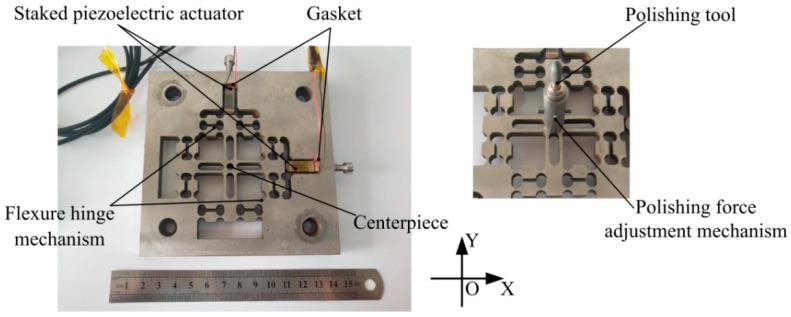
Structure of the decoupled non-resonant two-dimensional vibration-assisted polishing (2D-VAP) device.

The symmetric distribution is adopted in the flexure hinge mechanism, which the motion coupling error of the mechanism can be effectively avoided. Furthermore, the decoupling was verified through the offline performance test. On the basis of the existing device of the research group [29], the straight beam-type flexure hinge is improved to be the half corner-filleted flexure hinge, which has the advantages of high precision, large range of motion and greater turning ability. In addition, more precise motion of the flexure hinge mechanism can be obtained than with the previous device. The connection between the half corner-filleted flexure hinge and the central rigid platform is chamfered to prevent stress concentration and generating fatigue cracks. Shown in Figure 2 is the structural diagram of the decoupled non-resonant 2D-VAP device which actuated by two mutually perpendicular stacked piezoelectric actuators. The center is the polishing force adjustment mechanism, the polishing tool is installed on it. Moreover, polishing tools of different materials can be replaced as needed.

### 2.3. Stiffness Calculation

Stiffness calculation is one of the most important steps in flexure mechanism design. In order to generate decoupled and stable motion output at the polishing tool, the double parallelogram flexure hinge mechanism and symmetric distribution are adopted.

**Figure 3:**
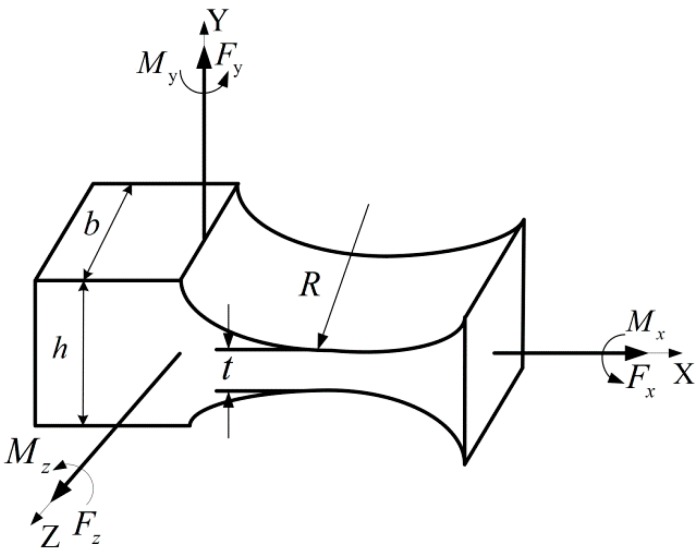
Parameters of the right circular flexure hinge.

The geometrical structure of the right circular flexure hinge (RCFH) is shown in Figure 3. The stem section of the hinge is rectangular, and the hinge is cut by two symmetric cylinders, which are perpendicular to the stem section. *R*, *t*, *b* and *h* are the cutting radius, the minimum thickness, the width and the height of the flexure hinge, respectively, where *h = t +* 2*R*. According to the rotation compliance of RCFH around the Z axis given by Paros, can be expressed as [30]:

(2) $$\frac{\alpha_{z}}{M_{z}}=\frac{3}{2EbR^{2}}\left[\frac{1}{2\beta +\beta^{2}}\right]\times \left\{\begin{array}{l} \left[\frac{1+\beta }{\gamma^{2}}+\frac{3+2\beta +\beta^{2}}{\gamma \left(2\beta +\beta^{2}\right)}\right]\left[\sqrt{1-{\left(1+\beta -\gamma \right)}^{2}}\right]+\\ \left[\frac{6\left(1+\beta \right)}{{\left(2\beta +\beta^{2}\right)}^{3/2}}\right]\left[\tan^{-1}\left[\sqrt{\frac{2+\beta }{\beta }}\times \frac{\left(\gamma -\beta \right)}{\sqrt{1-{\left(1+\beta -\gamma \right)}^{2}}}\right]\right] \end{array}\right\}$$

where $\beta =\frac{t}{2R}$, $\gamma =\frac{h}{2R}$, *E* is the Young’s modulus of the flexure hinge material.

In the meantime, the article also showed that when the minimum thickness *t* of the flexure hinge was far less than the height *h* and the cutting radius *R* of the flexure hinge, the above equation can be simplified as [30]:

(3) $$\frac{\alpha_{z}}{M_{z}}=\frac{9\pi R^{1/2}}{2Ebt^{5/2}}$$

**Figure 4:**
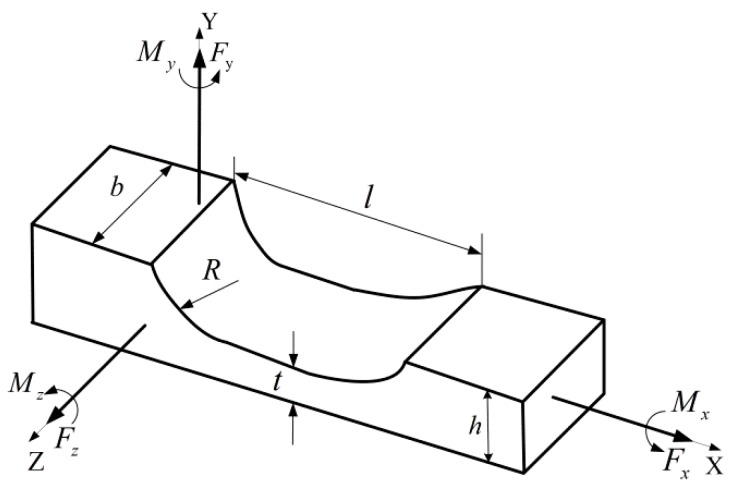
Parameters of the half corner-filleted flexure hinge.

The geometrical structure of the half corner-filleted flexure hinge is shown in Figure 4. The stem section of the hinge is rectangular, *t, h, b, R* and *l* are the minimum thickness, the height, the width, the fillet radius and the length of the flexure hinge, respectively. The rotation compliance of the flexure hinge under the action of torque *M_z_* can be expressed as [31]:

(4) $$C_{\alpha_{z,M_{z}}}=\frac{12}{Eb}\left[\frac{l-2R}{t^{3}}+\frac{R\left(3R^{2}+4Rt+2t^{2}\right)}{t^{2}\left(R+t\right){\left(2R+t\right)}^{2}}+\frac{6R^{2}\left(R+t\right)}{\sqrt{t^{5}{\left(2R+t\right)}^{5}}}\arctan\sqrt{1+\frac{2R}{t}}\right]$$

The flexure hinge mechanism generates a displacement in the X-direction or Y-direction under the unidirectional load *F*, each flexure unit generates an angular deformation under this action. It is assumed that the deformation of each part of the flexure hinges does not affect each other. In addition, the tensile deformation energy can thus be regarded as negligible compared with the rotational deformation energy in the deformation process. The connection between the half corner-filleted flexure hinges and the central rigid platform is chamfered to prevent stress concentration and generating fatigue cracks. However, the chamfer parts have little effect on the whole output placement, so the influence of these parts can be ignored in theory. According to the law of conservation of energy, the flexure hinge mechanism generates a displacement ΔL under the unidirectional load *F* in X-direction or Y-direction, which is expressed by *W*, and all of which is converted into rotational deformation energy $E_{r}$. As the flexure hinge mechanism in this paper is composed of right circular flexure hinges and half corner-filleted flexure hinges, the rotational deformation includes the rotational deformation of the right circular flexure hinges and the half corner-filleted flexure hinges.

(5) $$W=E_{r}$$

(6) $$W=\frac{1}{2}F\Delta L$$

(7) $$E_{r}=8\times \frac{1}{2}K_{\theta }\theta^{2}+4\times \frac{1}{2}K_{\alpha }\alpha^{2}$$

where ΔL is the displacement deformation generated under the unidirectional load *F*; $K_{\theta }$ is the rotational stiffness of RCFH; $K_{\alpha }$ is the rotational stiffness of the half corner-filleted flexural hinge. θ and α are the angular deformation of RCFH and half corner-filleted flexural hinge, respectively.

Since the output displacement ΔL is so small that it is far less than the length of the flexure hinge, the angular deformation in Equation (7) can be expressed as:

(8) $$\theta \approx \sin\theta =\frac{\Delta L}{l_{1}}$$

(9) $$\alpha \approx \sin\alpha =\frac{\Delta L}{l_{2}}$$

where $l_{1}$ is the vertical distance between the rotation axis of the compliant prismatic pair sub single-branch flexure hinge which composed of the right circle flexure hinges, $l_{2}$ is the length of the half corner-filleted flexure hinge.

Equation (5) can be expressed as:

(10) $$\frac{1}{2}F\Delta L=4K_{\theta }\frac{\Delta L^{2}}{l_{1}{}^{2}}+2K_{\alpha }\frac{\Delta L^{2}}{l_{2}{}^{2}}$$

Since the device is designed symmetrically, when the unidirectional load *F* is applied to the X- direction or Y-direction, the theoretical stiffness value of the flexure hinges along the motion direction can be expressed as:

(11) $$K_{x}=K_{y}=\frac{F}{\Delta L}=\frac{8K_{\theta }}{l_{1}{}^{2}}+\frac{4K_{\alpha }}{l_{2}{}^{2}}\approx 14.74\;N/\mu m$$

The theoretical stiffness of the flexure hinge mechanism which consists of right circular flexure hinges and half corner-filleted flexure hinges can be calculated by the above equation. The result is $K_{x}=K_{y}\approx 14.74\;N/\mu m$.

### 2.4. Numerical Simulation of Flexure Mechanism

In the process of polishing, the reciprocating deformation of the flexure hinge mechanism is achieved by the drive of the stacked piezoelectric actuators. Therefore, the internal stress is easily generated at the hinges. If the stress concentration in some parts of flexure hinges is too high, then the deformation and fatigue are likely to occur after a period of working process. Based on the theoretical analysis of the flexure hinge mechanism in the previous section, the performance parameters of the flexure hinge mechanism are analyzed using Workbench finite element software in this section. The material of the flexure hinge mechanism used in the simulations is 65Mn spring steel with good elasticity, for which the density is 7800 kg/m^3^; modulus of elasticity is 206 GPa, and Poisson’s ratio is 0.3. Table 1 shows the main parameters of 65Mn spring steel. Using Workbench software for simulation analysis, the first step is to establish the mechanism model. The steps are as follows:

- Set the material properties in the Workbench according to the parameter values in Table 1.
- The flexure mechanism geometry model was saved as .stp format and imported into the Workbench finite element software to generate finite element model.
- In the process of meshing the model, the results of finite element analysis are not ideal if the mesh is too sparse or too dense. Too sparse mesh will lead to low accuracy and inaccurate modeling result. If the mesh is too dense, the operation time will be increased and affect the work efficiency. Due to the complexity of the flexure hinge mechanism, Tetrahedrons meshing method and Patch Conforming algorithm were selected to mesh the model with which the meshes can be generated quickly and automatically. Furthermore, Tetrahedrons meshing method will be applicable to a variety of complex geometric shapes; Proximity and Curvature can be used in the key areas to refine the mesh automatically. Finally, the model was divided into 519,284 elements containing 792,475 nodes.

**Figure 5:**
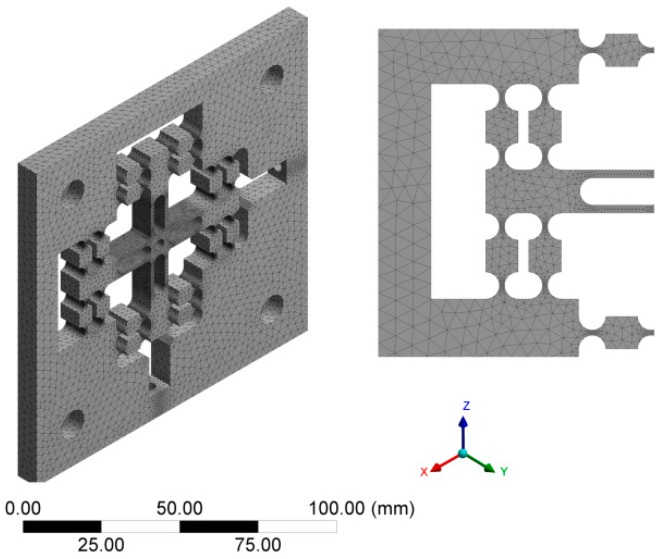
Finite element mesh model.

The final finite element model is shown in Figure 5, from which it can be seen that the key parts of the flexure hinge have been refined. However, the regular shape of the model was divided into sparse meshes, which not only improved the efficiency of software operation but also made the analysis result more accurate.

#### 2.4.1. Static Analysis of Flexure Mechanism

The statics analysis of the mechanism refers to the analysis of the displacement, stress and strain of the mechanism under the constant load without considering the influence of velocity and damping. Generally, the statics analysis of flexure hinge guide mechanism mainly includes stiffness analysis and stress analysis.

##### Stiffness Analysis

In order to compare the theoretical stiffness value with numerical simulation stiffness value and to ensure that the output displacement in the direction of the load can meet the performance requirements of the flexure hinges, it is necessary to carry out the stiffness simulation analysis. Since the flexure hinge mechanism is designed symmetrically, it is only necessary to select one direction for simulation. After completing the mesh generation according to the above steps, the boundary conditions need to be determined. In this paper, the four borders of the flexure mechanism were fixed. Subsequently, a load was applied to the appropriate contact point between the mechanism and the staked piezoelectric actuator to obtain a corresponding displacement.

**Figure 6:**
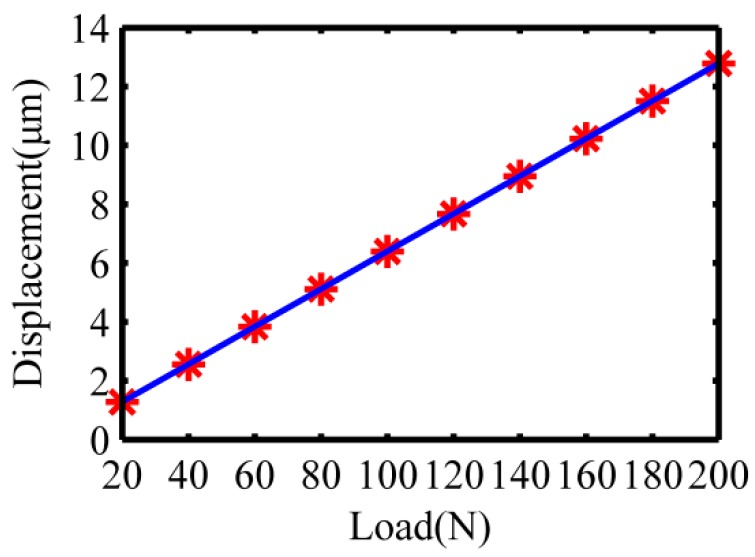
Fitting relationship between applied load and output displacement.

**Figure 7:**
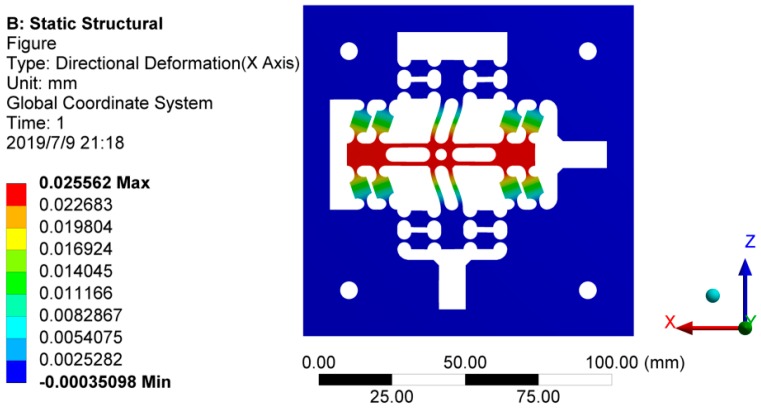
Force deformation diagram of flexure mechanism model.

A constant load *F* of 20–200 N is applied in the single direction at the mechanism’s point of contact with the stacked piezoelectric actuator to achieve the corresponding output displacements, 10 sets of loads and corresponding output displacements were selected as shown in Table 2. Matlab software was used to conduct the least squares fitting of 10 sets of the discrete data in Table 2, and a linear relationship between the applied load on the flexure hinge guide mechanism and the output displacement was obtained, as shown in Figure 6. The stiffness simulation result of the flexure hinge guide mechanism was calculated to be 15.65 N/μm, which shows slightly departure from the theoretical stiffness of 14.74 N/μm. The main reasons are that the rigid bodies outside the flexure hinges are all set as ideal rigid bodies, and the energy of tensile deformation of the flexure hinges is neglected in the theoretical calculation. In Workbench finite element simulation, the imported model was simplified to shorten the analysis time. Figure 7 shows the force deformation of the load along the X-direction of the flexure mechanism model. The moving end of the mechanism can effectively output a large displacement of 25.562 um, which is sufficient to meet the requirements of vibration-assisted polishing.

##### Stress Analysis

To ensure that the flexure hinge mechanism is in a safe state during polishing, it is necessary to perform static analysis to determine whether the maximum stress of it meets the stress limit of the material. The material of the flexure hinge mechanism is 65Mn spring steel of which the ultimate stress is 785 MPa. After heat treatment, the material has certain toughness and plasticity; the safety factor can be selected between 1.5 and 2.0. To ensure the safety and reliability of components under external forces, the safety factor *r* = 2 is selected to calculate the allowable stress [32]. The allowable stress of the material is expressed in Equation (12).

(12) $$\left[\sigma \right]=\frac{\left[\sigma_{s}\right]}{r}=392.5\;\mathrm{MPa}$$

**Figure 8:**
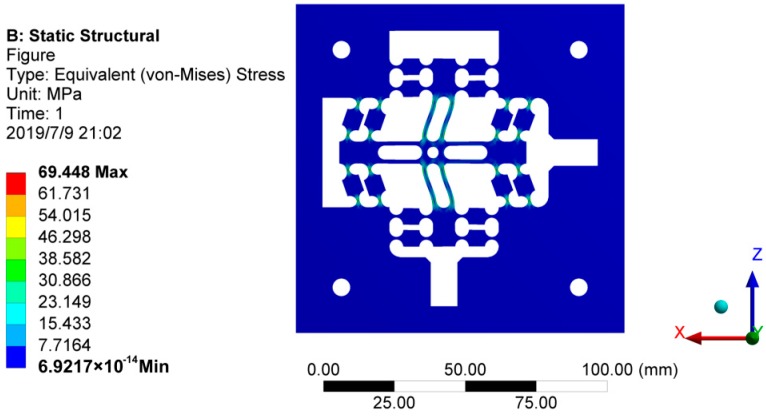
Stress cloud chart of flexure hinge mechanism under maximum working stroke.

Staked piezoelectric actuator (P885.1) produced by German PI Company is used to actuate the flexure hinge mechanism, and its maximum working stroke is 15 μm. When the influence of preload on staked piezoelectric actuator is not taken into account, the maximum stroke of the guide mechanism is 15 μm, then the maximum stress and stress distribution of the flexure hinge mechanism are analyzed using Workbench finite element software. The maximum stress is 69.448 Mpa under the above condition, which is far less than the allowable stress of 65Mn spring steel, as shown in Figure 8. Therefore, the flexure hinge mechanism is in a relatively safe working state during the whole process, which the material can be guaranteed to be in the elastic deformation range and thus achieve the accurate linear output displacement.

#### 2.4.2. Dynamic Analysis of Flexure Mechanism

**Figure 9:**
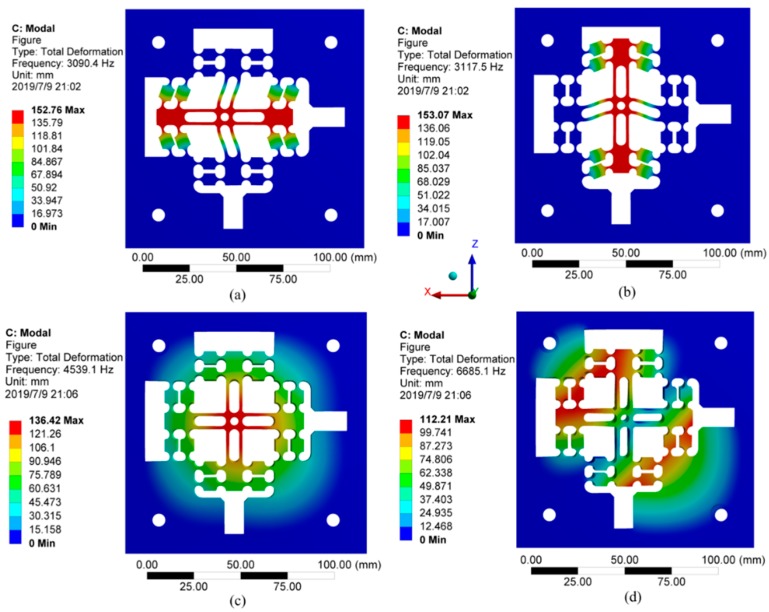
Modal analysis of the flexure hinges. (**a**) First-order modal (3090.4 Hz); (**b**) second-order modal (3117.5 Hz); (**c**) third-order modal (4539.1 Hz); (**d**) fourth-order modal (6685.1 Hz).

Modal analysis is the basis of dynamics analysis. The flexure hinge guide mechanism is driven by the high frequency driving force of the staked piezoelectric actuator during operation. In order to ensure that no resonance phenomenon occurs, the vibration mode and corresponding resonance frequency of each stage of the mechanism must be determined. The dynamic characteristics of the flexure hinge mechanism are analyzed using Workbench to achieve the first four modes. As shown in Figure 9, the natural frequencies of first and second order modes which along the X-direction and Z-direction are quite different from the natural frequency of third order mode, which is in accordance with an ideal working state. However, the experimental frequency is obviously lower than the first natural frequency, so it will not be affected by the natural frequency in actual work.

### 2.5. Measurement of the Vibration Trajectory Synthesized at the Polishing Tool

**Figure 10:**
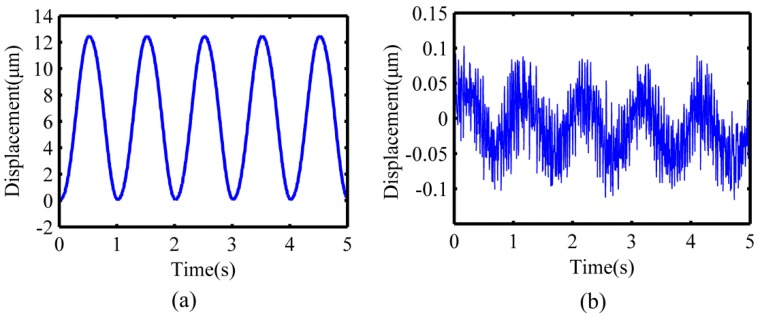
Motion test results in the X-direction. (**a**) Maximum output displacement in the X-direction; (**b**) motion coupling in the Y-direction.

**Figure 11:**
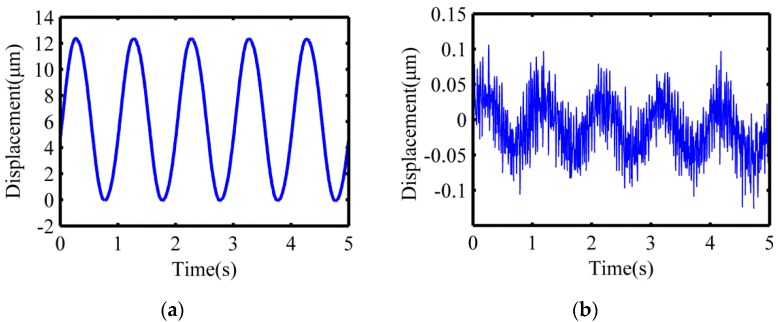
Motion test results in the Y-direction. (**a**) Maximum output displacement in the Y-direction; (**b**) motion coupling in the X-direction.

Before measuring the closed trajectory synthesized at the output end, the maximum stroke and the motion coupling of the flexure hinge mechanism in the X-direction and Y-direction should be tested first. The low-frequency harmonic signal of voltage 10 V and frequency 1 Hz were applied to excite the staked piezoelectric actuator to actuate the flexure hinges in the X-direction and Y-direction, respectively. The capacitive displacement sensors (Microsense TM II 5300, MicroSense, Lowell, MA, USA) were used to simultaneously collect the output displacement in the X-direction and Y-direction. As shown in Figure 10, the maximum output displacement in the X-direction is about 12.37 μm, and the motion coupling in the Y-direction is about 1.6% of the maximum stroke. Similarly, as shown in Figure 11, the maximum output displacement in the Y-direction is about 12.41 μm, and the motion coupling in the X-direction is about 1.8% of the maximum stroke. The experimental results show that the maximum stroke of the device in the X-direction and Y-direction are both more than 10 μm, and the corresponding motion coupling is less than 2% of the maximum stroke, which meet the requirements of the polishing experiment.

**Figure 12:**
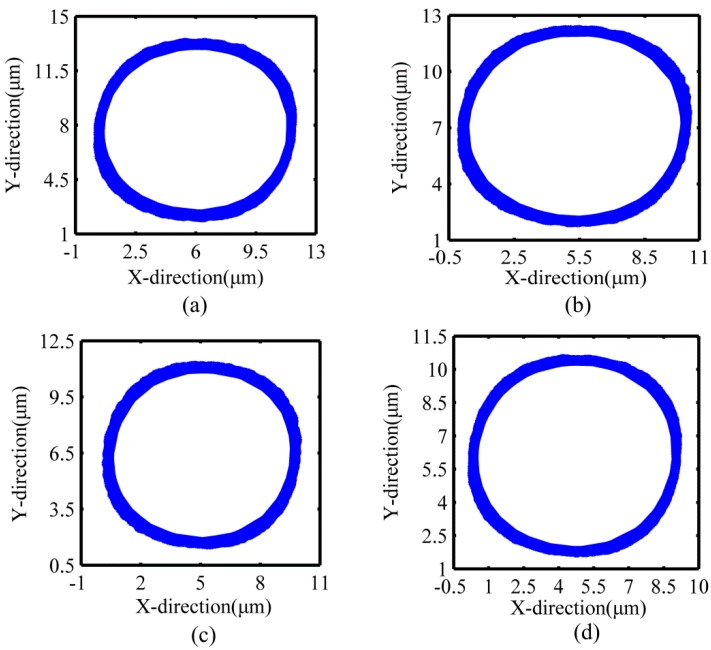
Measurement of the trajectory synthesized at the polishing tool. (**a**) Voltage: 10 V, $f_{1}=f_{2}=400\;\mathrm{Hz}$; (**b**) voltage: 9 V, $f_{1}=f_{2}=400\;\mathrm{Hz}$; (**c**) voltage: 8 V, $f_{1}=f_{2}=400\;\mathrm{Hz}$; (**d**) voltage: 7 V, $f_{1}=f_{2}=400\;\mathrm{Hz}$.

The natural frequency of the decoupled non-resonant 2D-VAP device is 726.8 Hz, which was obtained by striking with the force hammer (Kistler 9724A2000, Kistler, Winterthur, Switzerland), while the actual driving frequency of the staked piezoelectric actuator is usually 400 Hz, so it will not be affected by the natural frequency. The values of voltage and frequency shown in Figure 12 were applied to excite the staked piezoelectric actuators to actuate the flexure hinges in the X-direction and Y-direction simultaneously ($f_{1}=f_{2}$). The capacitive displacement sensors (Microsense TM II 5300) were used to simultaneously collect the output displacements in the X-direction and Y-direction to synthesize the closed motion trajectory. As shown in Figure 12a–d, the corresponding amplitude decreases obviously with the decrease of input voltage. It can also be seen that the circular closed motion trajectory at the output end is very close to the ideal circle, which indicates that the decoupled non-resonant 2D-VAP device has good decoupling.

### 2.6. Polishing Force Adjustment Mechanism

**Figure 13:**
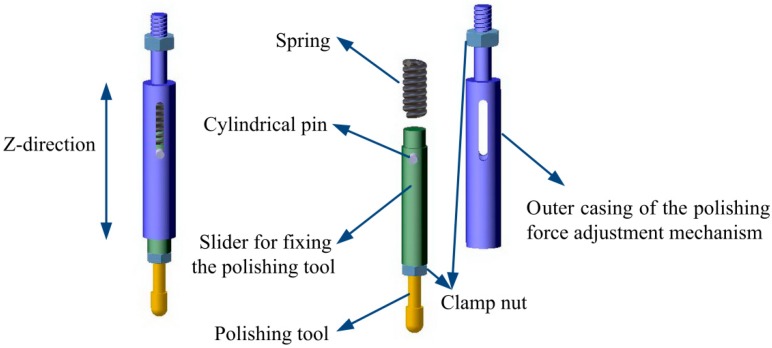
Polishing force adjustment mechanism.

The constant polishing force is the premise to ensure the removal function experiment. Therefore, it is necessary to design the polishing force adjustment mechanism, and the working principle is shown in Figure 13. The handle of the polishing tool is threaded and mounted on a slider for fixing the polishing tool, then fastened with a nut. The top of the slider is a boss which has the same diameter with the inner diameter of the spring, which prevents the spring from being displaced and is used to compress the spring. The cylindrical pin on the slider is for preventing the spring and the slider for fixing the polishing tool from being separated from the outer casing of the polishing force adjustment mechanism. The clearance fit is applied between the slider for fixing the polishing tool and the outer casing of the polishing force adjustment mechanism. Subsequently, the polishing tool can be moved in the Z-direction by compressing spring. The polishing force adjustment mechanism is fixed on the center of the flexure hinge mechanism; the polishing tool could be driven along the Z-direction. The distance between the polishing tool and the surface of the workpiece decreases gradually; when the two come into contact, the length of the spring changes from the original length l to $l^{\prime }$. The force applied on the surface of the workpiece can be calculated by Hooke’s law $F=k\times \left(l-l^{\prime }\right)$. The force gauge connected below the workpiece can be used to monitor the polishing force in real time and control the polishing force. Furthermore, constant force polishing or change the polishing force as required can be performed.

## 3. Material Removal Function

### 3.1. Theoretical Background

The most important characteristic of the 2D-VAP surface shaping technology is the deterministic control of material removal quantity, which is mainly reflected in the control of removal function. Therefore, the study of material removal function is very necessary. The material removal amount on the surface of the workpiece is achieved by convolution of the dwell time at different polishing points with removal function, as shown in Equation (13) [33].

(13) E(x,y)=R(x,y)∗∗T(x,y)

where E(x,y), R(x,y), T(x,y), ∗∗ represent the amount of material removal, removal function, dwell time of the polishing tool at (x,y) and convolution, respectively.

According to the Preston equation proposed by Preston in 1972 [34], it indicated that the material removal rate is proportional to the relative velocity and the pressure, as shown in Equation (14):

(14) $$\Delta h=k_{w}PV$$

where Δh is the amount of material removal per unit time; $k_{w}$ is the process coefficient; *P* is the pressure between the polishing tool and the workpiece; *V* is the instantaneous velocity of a point on the surface of the workpiece.

The removal function is achieved after the integral normalization of the Equation (14), which is expressed below [35]:

(15) $$R\left(x,y\right)=\underset{T\to \infty }{\lim}\frac{1}{T}k_{w}\int_{0}^{T}P\left(x,y\right)V\left(x,y\right)dt$$

where *T* is the total polishing time of point (x,y) within a polishing cycle; V(x,y) is the relative velocity between the polishing tool and the workpiece at the point (x,y); P(x,y) is the pressure at the point (x,y).

#### 3.1.1. Contact Pressure Between the Polishing Tool and the Workpiece

**Figure 14:**
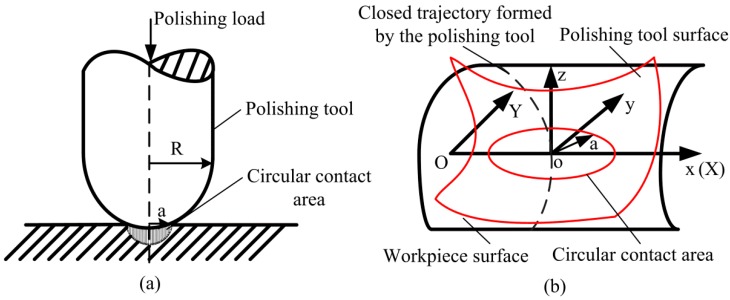
Surface contact schematic. (**a**) Schematic of polishing tool and workpiece surface to be polished; (**b**) schematic of contact area.

The Hertz contact theory is the theory of studying the local stress and strain distribution law of two objects after contacting each other due to pressure. According to the research, the theoretical analysis of the polished region was carried out by using the Hertz contact theory, and the hypothesis was verified by experiments [23]. The workpiece was flat and polished with a spherical polishing tool, as shown in Figure 14a. On the premise of satisfying the Hertz contact theory, since the radius of curvature of the spherical polishing tool was much smaller than the radius of curvature of the processing plane, the contact area could be approximately circular. Therefore, the formed polished contact area was represented as a circle with an approximate radius of *a*, as shown in Figure 14b.

(16) $$x^{2}+y^{2}=a^{2}$$

The pressure distribution in the contact circle area is:

(17) $$P\left(x,y\right)=-\frac{P_{0}}{a}\sqrt{a^{2}-x^{2}-y^{2}}$$

(18) $$P_{0}=\frac{3F_{N}}{2\pi a^{2}}$$

(19) $$a={\left(\frac{3F_{N}R_{e}}{4E^{*}}\right)}^{1/3}$$

(20) $$\frac{1}{E^{*}}=\frac{1-\mu_{1}{}^{2}}{E_{1}}+\frac{1-\mu_{2}{}^{2}}{E_{2}}$$

In the Equations (17)–(20), *P*_0_ is the pressure at the center point of the contact circle; *a* is the equivalent radius of the contact circle; *F_N_* is normal positive pressure; $E^{*}$ is the relative elastic modulus; *E*_1_ and *E*_2_, *μ*_1_ and *μ*_2_ are the elastic modulus and Poisson ratios of the polishing tool and the workpiece, respectively. *R_e_* is the equivalent radius of curvature for contact, which can be expressed below [36]:

(21) $$A+B=\frac{1}{2}\left(\frac{1}{R^{\prime }}+\frac{1}{R^{\prime \prime }}\right)=\frac{1}{2}\left(\frac{1}{R_{1}{}^{\prime }}+\frac{1}{R_{1}{}^{\prime \prime }}+\frac{1}{R_{2}{}^{\prime }}+\frac{1}{R_{2}{}^{\prime \prime }}\right)$$

(22) $$B-A=\frac{1}{2}{\left[\begin{array}{l} {\left(\frac{1}{R_{1}{}^{\prime }}-\frac{1}{R_{1}{}^{\prime \prime }}\right)}^{2}+{\left(\frac{1}{R_{2}{}^{\prime }}-\frac{1}{R_{2}{}^{\prime \prime }}\right)}^{2}\\ +2\left(\frac{1}{R_{1}{}^{\prime }}-\frac{1}{R_{1}{}^{\prime \prime }}\right)\left(\frac{1}{R_{2}{}^{\prime }}-\frac{1}{R_{2}{}^{\prime \prime }}\right)\cos\left(2\alpha \right) \end{array}\right]}^{1/2}$$

(23) $$R_{e}={\left(R^{\prime }R^{\prime \prime }\right)}^{1/2}=\frac{1}{2}{\left(AB\right)}^{-1/2}$$

where $R^{\prime }$ and $R^{\prime \prime }$ are defined as the principal relative radius of curvature; $R_{1}{}^{\prime }$ and $R_{1}{}^{\prime \prime }$ are the maximum and minimum curvature radii of the polishing tool at the surface contact point; $R_{2}{}^{\prime }$ and $R_{2}{}^{\prime \prime }$ are the maximum and minimum curvature radii of the workpiece surface at the surface contact point; α is the angle of orthogonal principal plane which at the surface contact point formed between the polishing tool and the workpiece surface.

#### 3.1.2. Relative Velocity Between the Polishing Tool and the Workpiece

The relative velocity referred in the removal function is the relative velocity between the polishing tool and the workpiece when the workpiece is static. The relative motion between the polishing tool and the workpiece in the X-direction and Y-direction can be expressed as Equation (1). The relative velocity in the X-direction and Y-direction can be expressed as:

(24) $$\left\{ \begin{array}{l} V_{x}=2\pi f_{1}A_{1}\cos\left(2\pi f_{1}t+\theta_{1}\right)\\ V_{y}=2\pi f_{2}A_{2}\cos\left(2\pi f_{2}t+\theta_{2}\right) \end{array}\right.$$

The resultant velocity can be expressed as:

(25) $$V=\sqrt{V_{x}{}^{2}+V_{y}{}^{2}}$$

When $\theta_{1}=0$ in the X-direction vibration expression, the phase difference between the X-direction and Y-direction is $\frac{\pi }{2}$, $f_{1}=f_{2}=f$ and $A_{1}=A_{2}=A$, Equation (25) can be expressed as:

(26)  V=2πfA

### 3.2. Numerical Simulation of Removal Function

#### 3.2.1. Theoretical Model of Removal Function

**Figure 15:**
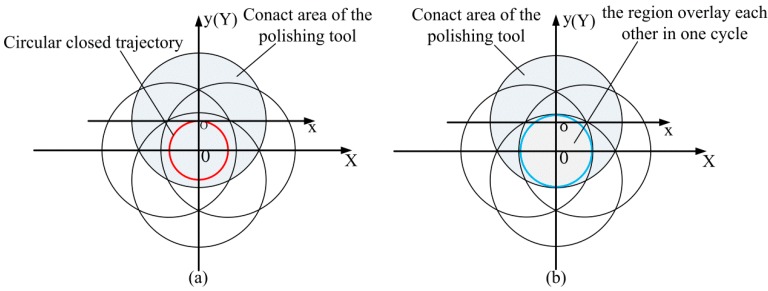
Schematic diagram of polishing. (**a**) Result of the polishing tool completing one cycle of vibration; (**b**) generation of the area which always contacts with the polishing tool throughout one vibration cycle.

**Figure 16:**
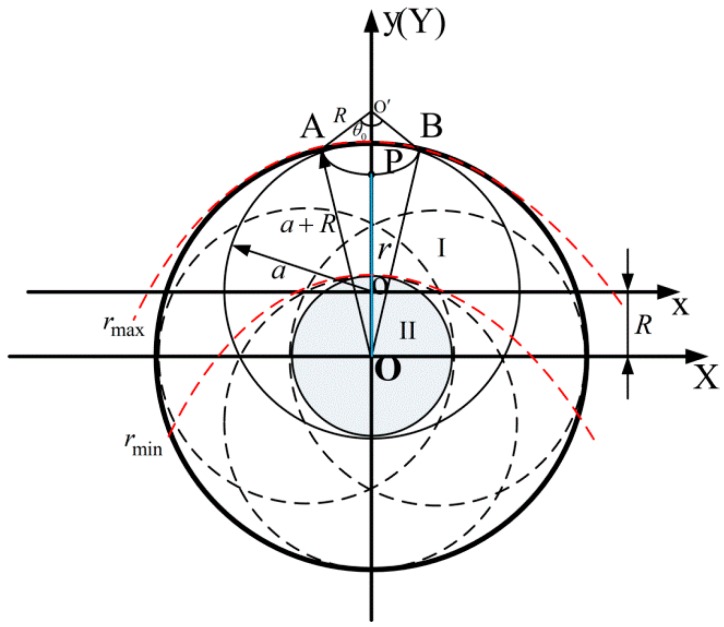
Schematic diagram of removal function.

The polishing tool vibrates according to the circular closed trajectory, that is, the center of the contact area formed by the contact between the polishing tool and the workpiece vibrates along the circular closed trajectory. Figure 15a is the result of the polishing tool completing one cycle of vibration. As shown in Figure 15b, a polishing area will be formed that, close to the center of the circular closed trajectory, always comes into contact with the polishing tool after one duty cycle. Figure 16 is the schematic diagram of the removal function obtained after vibrating according to the circular closed trajectory, where XOY is the workpiece coordinate system, and xoy is the polishing tool coordinate system.

Assuming that the radius of the circular closed trajectory is *R*, the radius of the contact area of the polishing tool is *a*, and the distance of any point P in the polishing area from the center O of the circular closed trajectory is *r*. Since *R < a* in actual manufacturing practice, the polishing area as shown in Figure 15 can be divided into two regions for analysis: When any point P in the polishing area belongs to the scope I, namely, $r_{\min}\leq r\leq r_{\max}$; when any point P in the polishing area belongs to the scope II, namely, $0\leq r\leq r_{\min}$; where $r_{\min}=a-R$, $r_{\max}=a+R$.

Transforming Equation (15) into polar coordinates can be expressed as:

(27) $$R\left(r,\theta \right)=\frac{1}{2\pi }k_{w}\int_{-\theta_{0}}^{\theta_{0}}P\left(r,\theta \right)V(r,\theta )d\theta$$

When any point P is in the scope I, an arc length with a center angle of $2\theta_{0}$ and a radius of *R* will be machined by polishing tool relative to the point P after a vibration cycle. The pressure distribution in the corresponding circular contact area is transformed into polar coordinates, which can be expressed as:

(28) $$P\left(r,\theta \right)=-\frac{P_{0}}{a}\sqrt{a^{2}-{\left(\left|\;o^{\prime }o\right|-R\cos\theta \right)}^{2}-{\left(R\sin\theta \right)}^{2}}$$

where $\left|o^{\prime }o\right|=\left|o^{\prime }O\right|-\left|\mathrm{oO}\right|=\left|o^{\prime }O\right|-R$, $\left|o^{\prime }O\right|=\left|\mathrm{PO}\right|+\left|o^{\prime }P\right|=\left|\mathrm{PO}\right|+R=r+R$.

Equation (28) can be expressed as:

(29) $$P\left(r,\theta \right)=-\frac{P_{0}}{a}\sqrt{a^{2}-{\left(r-R\cos\theta \right)}^{2}-{\left(R\sin\theta \right)}^{2}}$$

According to the cosine law, there are:

(30) $$\cos\theta_{0}=\frac{{\left(r+R\right)}^{2}+R^{2}-{\left(a+R\right)}^{2}}{2(r+R)R}$$

(31) $$\theta_{0}=\arccos\frac{{\left(r+R\right)}^{2}+R^{2}-{\left(a+R\right)}^{2}}{2(r+R)R}$$

Substitute Equations (26), (29), and (31) into Equation (27); the expression of the removal function for any point P in the scope I can be expressed as:

(32) $$R\left(r,\theta \right)=-\frac{1}{2\pi }k_{w}\frac{P_{0}}{a}\left(2\pi fA\right)\int_{-\arccos\theta_{0}}^{\arccos\theta_{0}}\sqrt{a^{2}-{\left(r-R\cos\theta \right)}^{2}-{\left(R\sin\theta \right)}^{2}}d\theta \;$$

When any point P is in the scope II, the point P is in contact with the polishing tool during a vibration cycle. An arc length with a center angle of 2π and a radius of *R* will be machined. In the same way, the expression of the removal function for any point P in the scope II can be expressed as:

(33) $$R\left(r,\theta \right)=-\frac{1}{2\pi }k_{w}\frac{P_{0}}{a}\left(2\pi fA\right)\int_{0}^{2\pi }\sqrt{a^{2}-{\left(r-R\cos\theta \right)}^{2}-{\left(R\sin\theta \right)}^{2}}d\theta \;$$

Through the above analysis, the removal function can be expressed as:

(34) $$R\left(r,\theta \right)=\left\{ \begin{array}{l} -\frac{1}{2\pi }k_{w}\int_{0}^{2\pi }P\left(r,\theta \right)V\left(r,\theta \right)d\theta \;,\left(0\leq r\leq r_{\min}\right)\\ -\frac{1}{2\pi }k_{w}\int_{-\arccos\theta_{0}}^{\arccos\theta_{0}}P\left(r,\theta \right)V\left(r,\theta \right)d\theta \;,\left(r_{\min}\leq r\leq r_{\max}\right) \end{array}\right.$$

#### 3.2.2. Modeling Results of Removal Function

**Figure 17:**
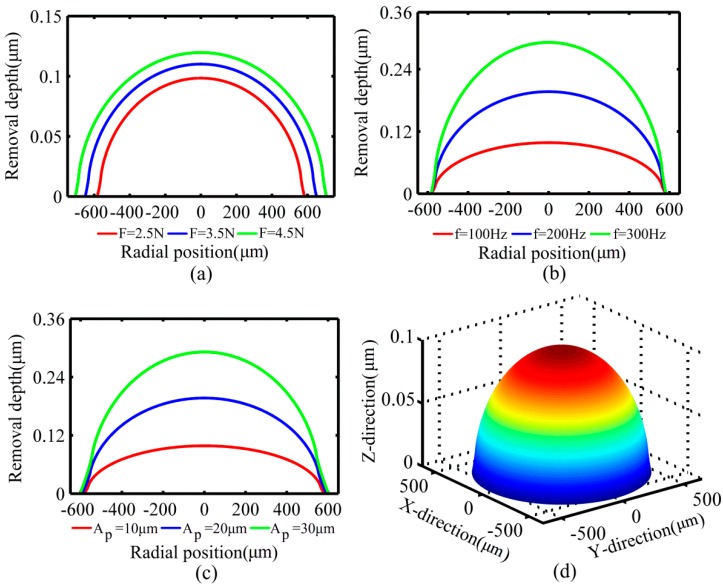
The modeling results of removal function with different parameters. (**a**) The effect of polishing force on the removal function; (**b**) the effect of vibration frequency on the removal function; (**c**) the effect of amplitude on the removal function; (**d**) three-dimensional view of the removal function.

According to Equation (34), the modeling results of removal functions with the parameters described below and comparative analyses are also carried out. To facilitate observation and comparison, the direction of modeling results of removal function is changed. Figure 17a shows a family of three modeling results of removal functions generated with increasing the polishing force, and with all other parameters fixed ($f_{1}=f_{2}=100\mathrm{Hz}$, $A_{1}=A_{2}=10\;\mu m$, $\Delta \theta =\frac{\pi }{2}$, radius of the polishing tool is 1 mm). The depth of material removal per unit time increases with the increase of polishing force. Figure 17b shows a family of three modeling results of removal functions generated with an increase of the vibration frequency and with all other parameters fixed ($f_{1}=f_{2}$, $A_{1}=A_{2}=10\;\mu m$, F=2.5 N, $\Delta \theta =\frac{\pi }{2}$, radius of the polishing tool is 1 mm). The depth of material removal per unit time increases with the increase of the vibration frequency. Figure 17c shows a family of three modeling results of removal functions generated with an increase of the vibration amplitude, and with all other parameters fixed ($A_{1}=A_{2}$, $f_{1}=f_{2}=100\;\mathrm{Hz}$, F=2.5 N, $\Delta \theta =\frac{\pi }{2}$, radius of the polishing tool is 1 mm). The depth of material removal per unit time increases with the increase of amplitude. Figure 17d shows the three-dimensional representation of the removal function.

## 4. Experiment of Removal Function

### 4.1. Experimental Preparation

**Figure 18:**
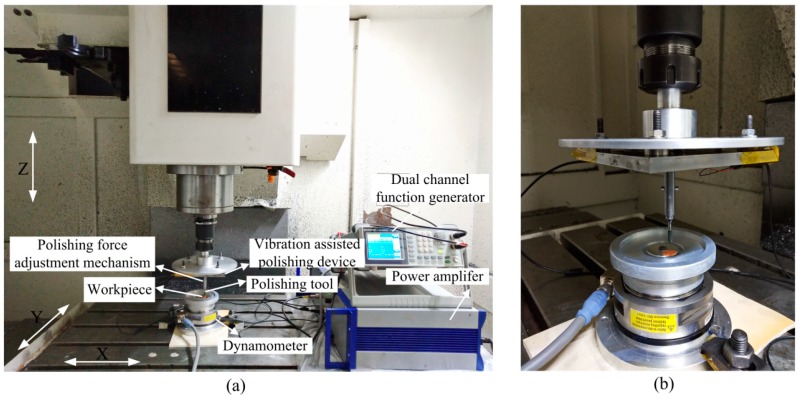
Experimental preparation. (**a**) Removal function experiment system. (**b**) Partial view.

The polishing system includes the polishing tool, polishing force adjustment mechanism, stacked piezoelectric actuators and flexure hinge mechanism, which are mounted on the Z-axis of the three-axis CNC milling machine. As shown in Figure 18a, the workpiece is fixed to the fixture by a screw, and a six-axis tool dynamometer (IP65 Delta Transducer, ATI, Apex, NC, USA) is attached to the fixture to measure the polishing force. A spherical silicone rubber polishing tool is used in this paper, which is a material with good elasticity. When it comes into contact with the surface of the workpiece, it has better conformality. In order to make the experiments of the removal function have a relatively significant effect, the diamond abrasive paste (W1.5) is adopted in the experiments. Before the experiment, the waveform is firstly observed by an oscilloscope to ensure the correctness of the dual channel signals. Sinusoidal voltages of varying magnitude and a phase shift of 90° are generated by a dual channel function generator and amplified by a dual channel signal-amplifier, finally transmitted to the stacked piezoelectric actuators to achieve the movement of the flexure hinge mechanism. Figure 18b is the partial view of the polishing system.

### 4.2. Results and Discussion

#### 4.2.1. Removal Function Profile

Decoupled non-resonant 2D-VAP belongs to deterministic removal processing, so the removal function is studied as one of the basic characteristics of deterministic removal. Single-point experiment method is adopted in removal function experiment, which means that the workpiece is fixed, and one or more removal function spots are processed at one or more places on the surface of the workpiece to obtain the removal function. The experiments of removal function were carried out by the vibration-assisted polishing system shown in Figure 18, and the experimental results were measured by white light interferometer (Zygo New View 8300, Zygo, Middlefield, CT, USA).

**Figure 19:**
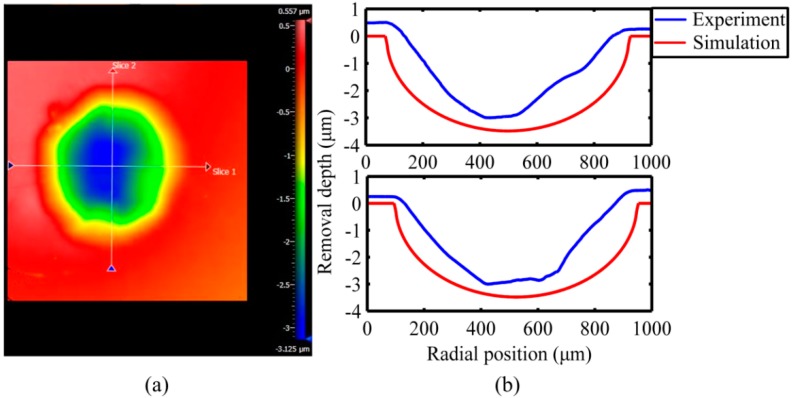
Measurement results. (**a**) Zygo measurement result of removal function; (**b**) comparison of theoretical model and experiment results.

The comparison result of the theoretical model and the experiment under the conditions in Table 3, are shown in Figure 19b; the modeling results have good coherence with experimental results. The maximum removal amount of the removal function is concentrated in the center, and both ends show a gradually decreasing trend distribution. Therefore, the shape of the removal function satisfies the demand of deterministic removal. Thereby the correctness of the theoretical model and the feasibility of the polishing system were verified. However, the modeling results are fuller than the experimental results, and there is no overlap between the two horizontal planes. The main reasons for the above situations are:

- A certain error existed when the flexure hinges were driven by stacked piezoelectric actuators, thus resulting in the error in the closed circular trajectory, and the removal function was also affected;
- In actual polishing, the relative velocity between polishing tool and workpiece was not exactly equal;
- The workpiece in the modeling result had an ideal smooth surface, and the ideal smooth surface of the actual workpiece was difficult to achieve, thus there was no overlap between the horizontal planes of the two results;
- The error was also caused by using the white light interferometer (Zygo New View 8300) for surface profile measurement.

#### 4.2.2. Stability Experiments of Removal Function

**Figure 20:**
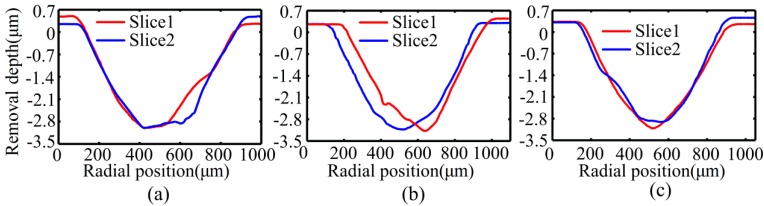
Results of partial single-point. (**a**) 2-D profile of the first point; (**b**) 2-D profile of the third point; (**c**) 2-D profile of the fifth point.

The stability of removal function was the premise to realize the deterministic removal of 2D-VAP; it is important to understand the trend of the removal function over time. In order to achieve the deterministic removal, it was necessary to ensure that the removal function in the process did not change greatly with time. Otherwise, there would be a great difference between the theoretical calculation and the actual removal amount, and the objective of quantitative removal could not be achieved. In order to study the stability of removal function, six single points were polished on the flat aluminum workpiece with the same polishing conditions as shown in Table 3. The single points were measured by white light interferometer (Zygo New View 8300), and the maximum material removal depth of single point after polishing and the polishing area were used as the standard to evaluate the stability of removal function, as shown in Table 4. Three of the single points were selected for comparison, as shown in Figure 20. The results of Table 4 and Figure 20 demonstrate that the removal function had good stability, which indicates that the polishing system proposed in this paper is suitable for deterministic removal processing.

## 5. Conclusions

In this paper, a new approach to decoupled non-resonant polishing apparatus which is directly driven by stacked piezoelectric actuators was proposed for vibration polishing on an aluminum workpiece. The following conclusions were obtained:

(a). The flexure mechanism is composed of right circular and half corner-filleted flexure hinges, which can achieve the high precision and large range of motion. In addition, the connection between the half corner-filleted flexure hinge and the central rigid platform is chamfered to prevent stress concentration and generating fatigue cracks. Stiffness modeling and finite element analysis were carried out for the flexure hinge mechanism, respectively. Through the comparison of theoretical stiffness and simulation stiffness, the correctness of the stiffness model was verified.

(b). Offline performance tests were carried out for the device, including the maximum stroke and motion coupling. The test results show that the maximum displacements of the device in the X-direction and Y-direction were 12.37 μm and 12.41 μm, respectively, and the amount of motion coupling in the X-direction and Y-direction was less than 2% of the maximum stroke, which met the requirements of the polishing experiment. The good motion decoupling of the flexure hinge mechanism was verified by the actual size of the trajectory synthesized at the polishing tool through the capacitive displacement sensors.

(c). According to the Preston equation, the theoretical model of the removal function when the polishing trajectory is circular was studied. The simulations of removal functions under different vibration parameters were carried out according to the theoretical model, and the maximum material removal depth per unit time was proportional to the vibration frequency, amplitude and polishing force, respectively. Furthermore, the comparison results of modeling and experiment show that both of them have similar shape, in which the maximum removal amount of the removal function is concentrated in the center, and both ends show a gradually decreasing trend distribution. Therefore, the shape of the removal function satisfies the demands of deterministic polishing. Finally, the feasibility of the polishing system proposed in this paper was verified through the removal function stability experiments, the results of which show that good stability is obtained. In addition, the shape of the removal function achieved in the single-point polishing experiment is conducive to the convergence of workpiece surface.

## Figures and Tables

**Table 1:** Main performance parameters of 65Mn spring steel.

Material	$\sigma_{s}/\mathrm{MPa}$	$\sigma_{b}/\mathrm{MPa}$	E/GPa	μ	G	$\rho \left({\mathrm{kg}/m}^{3}\right)$
65Mn	785	980	206	0.3	0.79	7800

**Table 2:** Load and displacement values.

Load (N)	20	40	60	80	100	120	140	160	180	200
Displacement (μm)	1.278	2.556	3.834	5.112	6.391	7.669	8.947	10.225	11.503	12.781

**Table 3:** Polishing condition.

Workpiece	Aluminum Alloy (6061)
Polishing tool	Silicone rubber
Radius curvature	1 mm
Abrasive	Diamond paste
Grain size	1.5 μm
Density	8.67%
Vibration mode	Circular
Vibration frequency	400 Hz
Vibration amplitude	4 μm
Polishing load	1 N
Polishing time	30 min

**Table 4:** Results of polishing area and the maximum removal depth values.

Fixed-Point Number	1	2	3	4	5	6	Average	Biggest Fluctuation
$\mathrm{Polishing}\;\mathrm{area}/{\mathrm{mm}}^{2}$	0.543	0.529	0.554	0.535	0.548	0.532	0.540	2.53%
maximum removal depth/μm	3.682	3.673	3.618	3.422	3.442	3.434	3.545	3.72%
